# The Effects of* Cordyceps sinensis* (Berk.) Sacc. and* Gymnema inodorum* (Lour.) Decne. Extracts on Adipogenesis and Lipase Activity* In Vitro*

**DOI:** 10.1155/2019/5370473

**Published:** 2019-04-01

**Authors:** Kanokwan Tiamyom, Kittipot Sirichaiwetchakoon, Tanaporn Hengpratom, Sajeera Kupittayanant, Rungrudee Srisawat, Atcharaporn Thaeomor, Griangsak Eumkeb

**Affiliations:** School of Preclinic, Institute of Science, Suranaree University of Technology, Nakhon Ratchasima 3000, Thailand

## Abstract

This study aimed to investigate the effects of* Cordyceps sinensis* extract (CSE) and* Gymnema inodorum* extract (GIE), used alone and combined, on antiadipogenesis in 3T3-L1 cells. Oil Red O staining was used to examine the effects of these extracts on inhibition of intracellular lipid accumulation in 3T3-L1 adipocytes and on lipid droplet morphology. Fourier transform-infrared (FTIR) microspectroscopy was used to examine biomolecular changes in 3T3-L1 adipocytes. The pancreatic lipase assay was used to evaluate the inhibitory effects of CSE and GIE on pancreatic lipase activity. Taken together, the results indicated that CSE, GIE, and their combination suppressed lipid accumulation. The FTIR microspectroscopy results indicated that CSE, GIE, and their combination had inhibitory effects on lipid accumulation in the adipocytes. Compared with the untreated adipocytes, the signal intensity and integrated areas of glycogen and other carbohydrates, the acyl chain of phospholipids, and the lipid/protein ratios of the CSE, GIE, alone, and combined treated adipocytes were significantly lower (*p* < 0.05). Combination treatment resulted in a synergistic effect on lipid accumulation reduction in the adipocytes. Principal component analysis of the biomolecular changes revealed six distinct clusters in the FTIR spectra of the sample cells. The pancreatic lipase assay results indicated that CSE and GIE inhibited the pancreatic lipase activity in a dose-dependent manner (mean ± standard error of the mean IC_50_ values, 2312.44 ± 176.55 *μ*g mL^−1^ and 982.24 ± 44.40 *μ*g mL^−1^, resp.). Our findings indicated that FTIR microspectroscopy has potential application for evaluation of the effectiveness of medicinal plants and for the development of infrared biochemical obesity markers useful for treating patients with obesity. These results suggested that use of CSE and GIE alone and in combination may be efficacious as a complementary therapy for hyperlipidemia and obesity management. However, clinical trials in animals and humans must first be completed.

## 1. Introduction

The incidence of obesity has been increasing steadily in developed and developing countries worldwide. Analysis of the global burden of obesity revealed that there were 396 million adults with obesity in 2005 and that the expected number is projected to be 573 million individuals in 2030, without adjusting for secular trends [1]. Excessive fat accumulation that increases the risk of adverse health effects is one definition of obesity. Obesity is implicated as a risk factor for various diseases (e.g., hypertension, coronary heart disease, and type II diabetes) [2, 3]. Despite the unavoidable progression of this disease and the positive effects of some medications on body weight reduction and alleviation of numerous cardiometabolic complications, large numbers of approved and wholesaled antiobesity drugs have been withdrawn due to serious adverse effects [4]. Phytochemicals present in plants traditionally used for medicinal purposes have the potential for use as newer therapeutics for obesity and other metabolic diseases [5].

Combinations of phytochemicals often occur naturally and using them in combination may significantly improve bioactivity.* Cordyceps sinensis* and* Gymnema inodorum* extracts are used as traditional food and medicine in Asia; they have received intense attention by a researcher in recent years [6–8]. Triterpenoids in* G. inodorum* leaves have an inhibitory effect on glucose absorption from the intestinal tract that relies on CH_2_OH production [7]. The potential hypoglycemic and renoprotective effects of* C. sinensis* solid-state fermented mycelia extract were examined in patients with type 2 diabetes mellitus [9]. Cordycepin inhibits adipocyte differentiation and accumulation of lipid in mature adipocytes. As cordycepin blocks both adipocyte differentiation and lipid accumulation, it has the potential to be an effective therapeutic agent for obesity and obesity-related disorders [10].

Synergy research has found that standardized phyto-drugs have therapeutic equivalence to the standard drugs. Compared with synthetic drugs, they also have fewer or no side effects [11]. However, the synergistic effects of* C. sinensis* extract (CSE) plus* G. inodorum* extract (GIE) on antiadipogenesis in 3T3-L1 adipocytes have not been investigated. The objective of this study was to examine the synergistic effects of CSE plus GIE on antiadipogenesis in 3T3-L1 cells.

## 2. Materials and Methods

### 2.1. Reagents and Cells

All chemicals were of the highest quality available (e.g., analytical grade). Bovine calf serum and 3T3-L1 mouse embryonic fibroblasts and were purchased from the American Type Culture Collection (ATCC, USA). Dulbecco's modified Eagle's medium (DMEM), penicillin, streptomycin, fetal bovine serum (FBS), 3-(4,5-dimethylthiazol-2-yl)-2,5-diphe-nyltetrazolium bromide (MTT), and N-2-hydroxyethylpiperazine-N-2-ethane sulfonic acid were obtained from Gibco Invitrogen (Grand Island, NY, USA). Insulin solution (bovine), 3-isobutyl-1-methylxanthine (IBMX), 4-nitrophenyl dodecanoate (pNP laurate), porcine pancreas lipase, and simvastatin (SIM) were obtained from Sigma-Aldrich (St. Louis, USA). Dimethyl sulfoxide (DMSO) was purchased from Carlo Erba Reagents S.r.l. (Chaussée du Vexin, Val de Reuil, USA). Dexamethasone was obtained from G Bioscience (St. Louis, USA). Oil Red O was purchased from Amresco Inc. (Solon, OH, USA).

### 2.2. Preparation of Plant Extracts

The* C. sinensis *extract was obtained from the Cordythai Company, Ltd., Thailand. The identity of the specimen was authenticated by a microbiologist at Kasetsart University (Bangkok, Thailand). Leaves of* G. inodorum *were collected from the Chiangda organic company garden (Chiang Mai, Thailand). These plant specimens were authenticated by Dr. Santi Wattana, a lecturer and plant biologist at the Institute of Science, Suranaree University of Technology. A specimen was deposited in the botanical garden, Suranaree University of Technology.

A 250-g dried powder of* G. inodorum *was soaked in 750 mL 95% ethanol at room temperature (25 ± 1°C) for 7 days with occasional stirring. After 7 days, the ethanol extract was filtered (Whatman no. 1 filter paper) and concentrated using a rotary evaporator at reduced pressure and <50°C. The concentrated extract was collected in a petri dish and air-dried to the point of complete evaporation of the ethanol. The blackish-green semisolid extract was kept at -20°C until use [12].

### 2.3. Preliminary Qualitative Phytochemical Screening Tests

The preliminary qualitative phytochemical screening analysis was performed as previously described, with some modification [13–15]. The chemical compounds of extract constituents (i.e., tannins, saponins, alkaloids, flavonoids, steroids, and glycosides) were tested.

### 2.4. Quantitative Estimation of Chemical Constituents

#### 2.4.1. Total Phenolic Content

The Folin–Ciocalteu assay was used to investigate the total phenolic content as previously described by Singleton et al. [16] and Rupasinghe et al. [17], with little modification. Briefly, 100 *μ*L 0.2 M Folin–Ciocalteu, 20 *μ*L CSE or GIE, and various concentrations (0.0025, 0.005, 0.0075, 0.01, 0.015, 0.02, and 0.0625 mg mL^−1^ in 100% methanol) of gallic acid were added to each well of 96-well plates. Then 80 *μ*L 7.5% (w/v) sodium carbonate was added to each well and the samples were incubated for 2 h at room temperature. The absorbance of the resulting blue color solution was measured using a spectrophotometer (wavelength, 765 nm). A gallic acid standard curve was used to determine the total phenolic content. The results were expressed as mg gallic acid equivalents (mg GAE/g) per g of dry weight.

#### 2.4.2. Total Flavonoid Content

The total flavonoid content was measured using the aluminium chloride colorimetric assay following Chen et al.'s and Settharaksa et al.'s methods [18, 19], with little modification. Briefly, 125 *μ*L distilled water, 25 *μ*L standard catechin at various concentrations (0.025, 0.05, 0.1, 0.2, 0.3, and 0.4 mg mL^−1^) or CSE or GIE, and 10 *μ*L 5% NaNO_2_ were mixed in each well of 96-well plates. The samples were then incubated for 6 min at room temperature and 15 *μ*L 10% AlCl_3_ solution was added to each well. The plates were incubated for 5 min at room temperature and then 50 *μ*L 1 M NaOH was added. Each plate was shaken in a microplate reader spectrophotometer for 5 min before measuring absorbance (wavelength, 595 nm). A catechin standard curve was used to determine the total flavonoid content. The results were presented as mg catechin equivalents (mg CE/g) per gram of dry weight.

### 2.5. Cell Culture

Phitaktim et al.'s methods with some modifications were used for 3T3-L1 preadipocyte culture and differentiation [20]. The culture medium was high-glucose DMEM supplemented with 10% bovine calf serum, 1.5 mg mL^−1^ sodium bicarbonate, 100 U mL^−1^ penicillin, and 100 mg mL^−1^ streptomycin; the cells were grown until being confluent. The incubation conditions were 37°C, 5% CO_2_, and 95% humidity. At 2 days after confluence was observed (day 0), the preadipocytes were induced to differentiate into adipocytes using 48-h culture in a differentiation medium containing 10% FBS, 1.0 mM dexamethasone, 0.5 mM IBMX, and 1.0 mg mL^−1^ insulin in DMEM. At 48 h (i.e., day 2), the differentiation medium was changed to a maintenance medium (10% FBS and 1.0 mg mL^−1^ insulin in DMEM) for another 48-h culture period (day 4). Every 48 h until day 10, the medium was replaced with newly-prepared maintenance medium. The preliminary concentration interval testing of CSE and GIE alone and combined to determine that the extracts were not toxic (i.e., not significantly different from the control) was then performed. This testing found that the dosage intervals of CSE at 10–40 *μ*g mL^−1^, GIE at 500–2000 *μ*g mL^−1^, and CSE plus GIE (CSE/GIE) at 10 + 500 *μ*g mL^−1^ were the correct intervals for the experiments. The SIM dosage was 1.67 *μ*g mL^−1^, which was the dosage used by Sirichaiwetchakoon et al. [21]. SIM was used as a positive control. During testing of preadipocyte differentiation, the cells were treated with the various obtained concentrations of CSE, GIE, and SIM alone, and with the CSE/GIE combination, for 48-h differentiation phase periods (i.e., at days 0, 2, 4, 6, and 8). The rates of differentiation into adipocytes were calculated on day 10.

### 2.6. In Vitro Cytotoxic Test (MTT Assay)

A tetrazolium dye (MTT) colorimetric assay was used to examine the cytotoxic effects of exposure to CSE, GIE, SIM alone, and CSE/GIE combination on cell proliferation [22]. The 96-well plates were seeded at a density of 5 × 10^3^ cells/well. Cell adherence was then allowed to progress for 48 h. The cells were then subjected to a 48-h exposure of various concentrations of four compounds. The medium was then removed, and the final concentration of 0.5 mg mL^−1^ MTT was added to each well. The cells were then incubated for 4 h at 37°C. DMSO was then used to dissolve the formazan crystals formed by viable cells and a microplate spectrophotometer (Bio-Rad Laboratories, Inc., USA) was used to measure the absorbance (wavelength, 540 nm). Linear regression analysis and a dose-response curve were used to calculate the extract concentration resulting in the death of 50% of the test cells (i.e., 50% lethal concentration (LC50)).

### 2.7. Oil Red O and Hematoxylin Staining

On day 10, Jarinyaporn et al.'s and Sirichaiwetchakoon et al.'s [21, 23] methods (including Oil Red O staining), with some modifications, were used to determine the rates of differentiation of preadipocytes into adipocytes that were associated with the amount of lipid accumulation. Briefly, adipogenesis of the 3T3-L1 preadipocytes was first induced using adipogenic medium; they were treated with concentrations similar to the established safe MTT concentration intervals of CSE at 10–40 *μ*g mL^−1^, GIE at 500–2000 *μ*g mL^−1^, SIM at 1.67 *μ*g mL^−1^, and CSE/GIE at 10 + 500 *μ*g mL^−1^ for 48 h. The differentiation medium was then changed to maintenance medium that included the treatment agents; this change in maintenance medium was then performed every 48 h until day 10. PBS was then used to wash the cells (twice) and then they were fixed for 1 h using formaldehyde (10%) in PBS. Distilled water was then used to wash the cells (twice) and they were stained using a 5% Oil Red O solution in 60:40 (v/v) isopropanol:distilled water at room temperature for 30 min. The Oil Red O-stained cells were then washed with distilled water (twice) and stained with hematoxylin solution at room temperature for 10 min. An inverted fluorescence microscope (Olympus Corporation, Japan) was used to examine the stained cells. Quantification of lipid accumulation was performed after washing the Oil Red O-stained triglyceride droplets with 60% isopropanol (twice), eluting with 100% isopropanol and transferring to a new 96-well plate. A microplate spectrophotometer was used to measure absorbance (wavelength, 490 nm). The equation (OD_sample_ −OD_blank_)/(OD_positive  control_ −OD_blank_) × 100 was used to calculate the differentiation rate (%).

The fractional effective concentration (FEC) index was calculated for each combination to measure the interaction between two agents. The FEC for each agent was calculated by dividing the concentration of the compound present in that treated group in combination where the effective concentration (EC) of the treated group showed equal or higher (or better) effect than a negative control group and those compounds treated alone. The FEC index (FECI) was calculated using the following: FEC of A compound = EC of A in combination/EC of A alone; FEC of B compound = EC of B in combination/EC of B alone; hence, FECI = FEC of A compound + FEC of B compound. When the FECI of the combination was <1.0, a combination was designated as synergistic. An FECI = 1.0 indicated “no interaction or zero-interaction” between the agents, and a value >1.0 indicated antagonism between the two compounds [24, 25].

### 2.8. Pancreatic Lipase Assay

The pancreatic lipase assay was used to investigate the effects of CSE and GIE on the reduction of fat digestion and absorption. Guo et al.'s and Hengpratom et al.'s [26, 27] methods, with slight modifications, were used to measure lipase activity. The supernatant of 5 mg mL^−1^ porcine pancreas lipase type 2 in distilled water was used for the assay. The reaction buffer consisted of 0.1% (w/v) pNP laurate buffer (100 mM Tris buffer pH 8.2) and 5 mM sodium acetate (pH 5.0) containing 1% Triton X-100. The buffer was heated in boiling water for 2 min and then cooled to room temperature. Ranges in preliminary concentrations of CSE, GIE were then tested to determine the concentrations appropriate for the calculation of the IC_50_ for lipase inhibitory activity. The concentration intervals of CSE at 1000–1600 *μ*g mL^−1^ and GIE at 250–1000 *μ*g mL^−1^ were obtained, which were almost similar to the ranges for Oil Red O staining. Fifty percent DMSO in reaction buffer was used to dissolve each test sample. The sample (20 *μ*L) and lipases (30 mL) were added to 40 mL reaction buffer; a substrate solution (30 mL) was used to initiate the reaction. The negative control contained 50% DMSO instead of the sample. Each mixture was incubated at 37°C for 2 h. A microplate spectrophotometer (wavelength, 405 nm) was used to measure absorbance. The inhibition rate (%) was calculated as (1−(OD_sample_ −OD_sample  blank_)/OD_negative  control_) × 100.

### 2.9. Focal Plane Array-FTIR Microspectroscopy

FTIR measurement and Siriwong et al.'s method with some modifications were used to evaluate the effects of CSE, GIE, and the CSE/GIE combination on the adipocytes [28]. The lowest concentrations of CSE (10 *μ*g mL^−1^) and GIE (500 *μ*g mL^−1^) used alone, and CSE/GIE (10 + 500 *μ*g mL^−1^) in combination, which the MTT assay revealed to be nontoxic and the Oil Red O staining indicated to have synergistic activity, were used to investigate biomolecular changes in the adipocytes (focal plane array-FTIR microspectroscopy). A positive control using SIM (1.67 *μ*g mL^−1^) at the same concentration as the MTT and Oil Red O assays was also tested. Preadipocytes, untreated adipocytes, and adipocytes treated using these agents at the above concentrations on day 10 were collected and centrifuged (4000 × g, 5 min). The cells were then washed with 0.85% NaCl (once) and recentrifuged (4000 × g, 5 min). The cell pellets were dropped onto a barium fluoride optical window 13 mm *Ø* × 2 mm ( Crystran, Ltd.). Excess water was removed using air drying. The dried cells were kept in a desiccator until FTIR analysis. For the FTIR microspectroscopy, each sample was evaluated in triplicate. Staff at a spectroscopy facility (Synchrotron Light Research Institute, Public Organization, Thailand) recorded the FTIR spectra using a Bruker Tensor 27 spectrometer (Globar source) coupled with a Bruker Hyperion 3000 microscope (Bruker Optics Inc., Ettlingen, Germany). The microscope was equipped with a 64 × 64 element mercury cadmium telluride, focal plane array detector, which allowed simultaneous acquisition of the spectral data. The FTIR samples were examined using the 15× objective, and the results of 64 scans were recorded in transmission mode at a 4 cm^−1^ spectral resolution. An 8 × 8 binning FTIR image mosaic was constructed from the images. A spectral range of 4000–700 cm^−1^ was used to acquire the absorbance spectra; the single spectra were acquired from approximately 20 *μ*m × 20 *μ*m sample areas. OPUS 7.2 software (Bruker Optics Ltd., Ettlingen, Germany) was used to obtain the FTIR spectral data and to control the instrument system. Microcal TM origin 6.0 software (Microcal Software, Inc., Northampton, USA) was used to plot the mean values for the spectral data as a stacked view. Principal component analysis (PCA) was used to identify sample spectra variability and clustering (Unscrambler X 10.1 software, CAMO Software AS, Oslo, Norway). During spectral preprocessing, second derivative transformations were obtained using Savitzky-Golay algorithms (nine smoothing points) and were normalized using an extended multiplicative signal correction (spectral regions, 3000–2800 cm^−1^ and 1800–950 cm^−1^). This method was used to identify regions of the absorption peak overlap, to reduce variation between replicate spectra and to correct for baseline shift. Score plots (2D) and loading plots were used to represent the different data classes and the associations between data set variables, respectively. The samples' signal intensities and integrated peak areas were analyzed using OPUS 7.2 software (Bruker). The results for the signal strengths and integrated peak areas of the lipids (3000–2800 cm^−1^), nucleic acids, and C-O vibrations from glycogen and other carbohydrates (1300–950 cm^−1^) were illustrated using a histogram.

### 2.10. Statistical Analysis

All results were expressed as a mean ± standard error of the mean (SEM) values. The statistically significant differences between treatment and control groups for cell viability, amounts of lipid accumulation, and biomolecular changes were analyzed using one-way analysis of variance and Tukey's honestly significant difference post-hoc test. Student's t-tests were used to determine statistically significant between-group differences in lipase inhibition activity. The results were considered statistically significant when* p* < 0.05 and were representative of at least three independent experiments [26, 29].

## 3. Results

### 3.1. Preliminary Phytochemical Analysis

Each lyophilized ethanol extract of* G. inodorum* was weighed to calculate the percentage yield of the extract. The percentage of yield obtained was 17.43% (w/w). The results of the preliminary qualitative phytochemical screening tests of* C. sinensis* and* G. inodorum* indicated that they contained flavonoids.* C. sinensis* contained alkaloids, tannins, and saponin and* G. inodorum* contained terpenoids and glycoside. Steroids were not found in extracts from either plant (Table 1). The total mean flavonoid content of* C. sinensis* and* G. inodorum* was 8.79 ± 2.46 and 4.99 ± 0.63 mg CE/g of dry weight, respectively. The total phenolic content of* C. sinensis* and* G. inodorum* was 0.14 ± 0.01 and 0.81 ± 0.01 mg GAE/g of dry weight, respectively.

### 3.2. 3T3-L1 Preadipocyte Viability Assay

**Figure 1:**
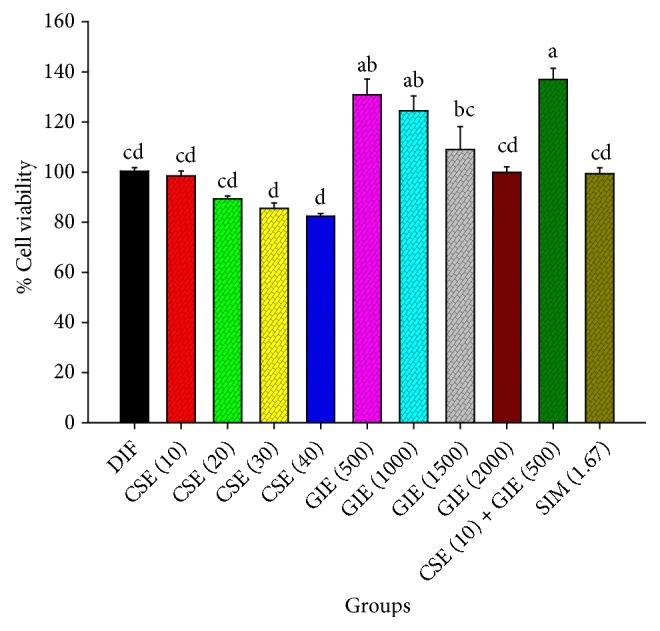
Effects of* C. sinensis* extract (CSE) and* G. inodorum* extract (GIE) used alone and combined on 3T3-L1 preadipocyte viability. DIF = differentiated adipocytes; CSE (10) = CSE at 10 *μ*g mL^−1^; GIE (500) = GIE at 500 *μ*g mL^−1^; CSE (10) + GIE (500) = CSE at 10 *μ*g mL^−1^ plus GIE at 500 *μ*g mL^−1^. SIM (1.67) = simvastatin at 1.67 *μ*g mL^−1^. Each value is the mean ± SEM of three (replicate) experiments and is expressed as population growth. The results are expressed as mean ± SEM values. Mean values followed by the same superscript letter were not significantly different (*p* < 0.05, Tukey's test).

The effects of CSE and GIE on preadipocyte viability were dose dependent (Figure 1). The LC_50_ values for the CSE and GIE antiproliferative effects were 112.23 ± 0.49 *μ*g mL^−1^ and 3075.73 ± 274.24 *μ*g mL^−1^, respectively. Assessment of the viability of the 3T3-L1 cells revealed that the CSE-treated group was not significantly different from the untreated group of preadipocytes. The GIE (500–1000) and CSE/GIE combination resulted in significantly greater cell viability than the untreated preadipocyte groups (*p *< 0.05). The nontoxic concentrations of CSE, GIE, and the CSE/GIE combination were chosen for further investigation using Oil Red O staining.

### 3.3. Effects of CSE, GIE, and the CSE/GIE Combination on Lipid Accumulation during Adipogenesis

**Figure 2:**
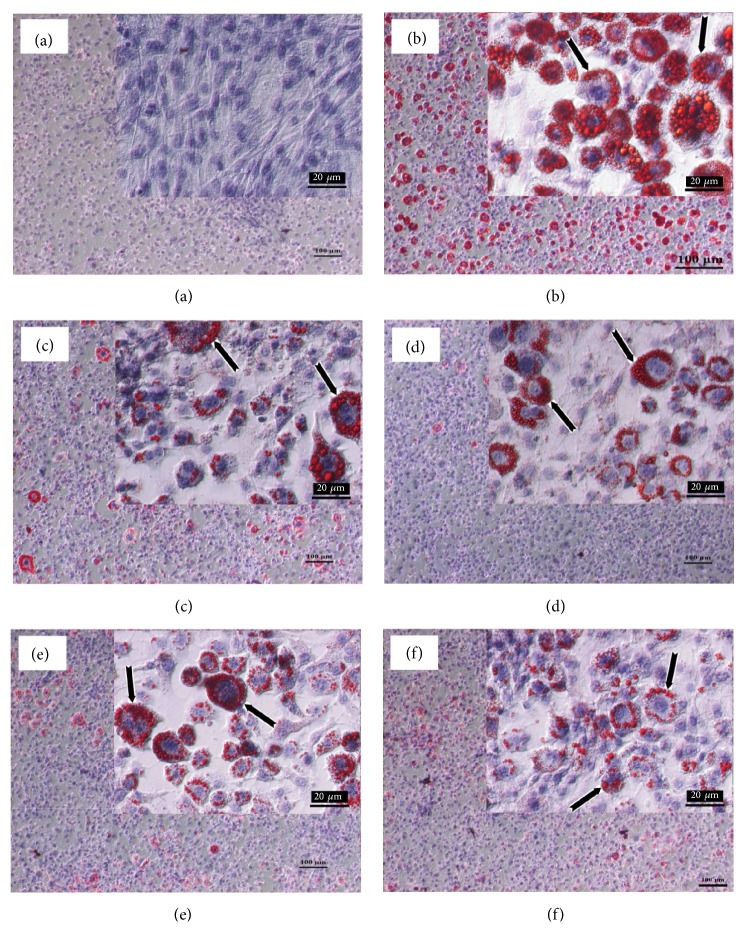
Results of Oil Red O and hematoxylin staining for intracellular lipid for six sample groups: (a) preadipocytes (nondifferentiated cells); (b) untreated adipocytes, (c) simvastatin at 1.67 *μ*g mL^−1^; (d) CSE at 10 *μ*g mL^−1^; (e) GIE at 500 *μ*g mL^−1^; (f) combination of CSE at 10 *μ*g mL^−1^ plus GIE at 500 *μ*g mL^−1^ (original magnification* x *100, scale bar: 100 *μ*m;* inset*:* x* 600, scale bar: 20 *μ*m.)

**Figure 3:**
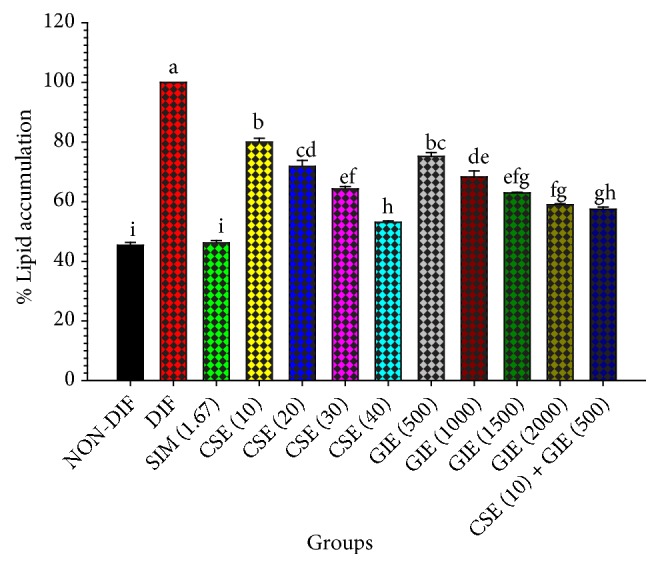
Graphical representation of the effects of CSE and GIE alone and combined on the percentage of intracellular lipid accumulation in differentiated 3T3-L1 cells, after Oil Red O staining. NON-DIF = preadipocytes (nondifferentiated cells); DIF = differentiated adipocytes (untreated adipocytes); SIM (1.67) = simvastatin at 1.67 *μ*g mL^−1^; CSE (10) = CSE at 10 *μ*g mL^−1^; GIE (500) = GIE at 500 *μ*g mL^−1^; the mean ± SEM values presented are for three replicates. Mean values followed by the same superscript letter were not statistically different (*p* < 0.05, Tukey's test).

The effects of CSE, GIE, and the CSE/GIE combination on lipid accumulation during 10 days of preadipocyte to adipocyte differentiation were examined. The results for microscopic examination of the Oil Red O and hematoxylin-stained cells indicated that exposure to CSE and GIE resulted in decreased numbers of Oil Red O-stained droplets in mature adipocytes at 10 and 500 *μ*g mL^−1^, respectively (Figure 2). The adipocyte intracellular lipid accumulation for the treated groups of CSE and GIE alone and combined was significantly decreased compared with untreated adipocytes (*p* < 0.05, Figure 3). The interaction of CSE (10) plus GIE (500) was compared to treating with only CSE or GIE. The EC for CSE (30) alone = 30 and the EC for CSE (10) in combination = 10. Therefore, the FEC for the CSE compound = 0.33. The EC for GIE (2000) alone = 2000 and the EC for GIE (500) in combination = 500. Therefore, the FEC for the GIE compound = 0.25. Thus, the FECI of the combination was 0.58 (Figure 3). These results suggested that the combination had a synergistic effect on the reduction in intracellular lipid accumulation. Intracellular fat deposit formation for SIM exposure at 1.67 *μ*g mL^−1^ was significantly reduced by 54 ± 1.37%, compared with the untreated adipocytes (*p* < 0.05) (Figure 3).

### 3.4. Effects of CSE and GIE on Pancreatic Lipase Activity

**Figure 4:**
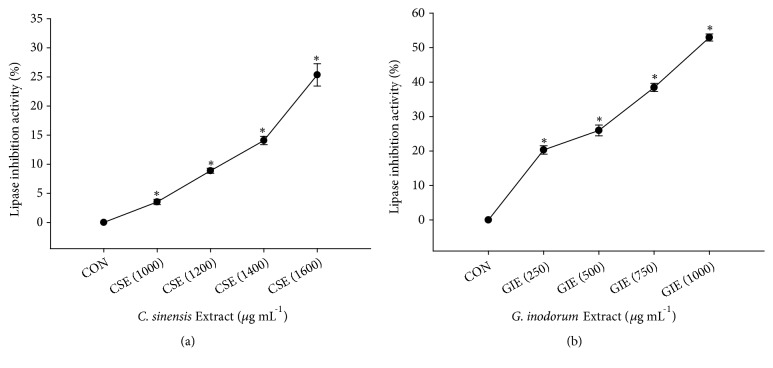
Inhibitory effects of CSE (a) and GIE (b) at various concentrations on pancreatic lipase activity (%). CON = control; CSE (1000) = CSE at 1000 *μ*g mL^−1^; GIE (250) = GIE at 250 *μ*g mL^−1^. Results are expressed as mean ± SEM values (three replicates). *∗ p *< 0.05 indicates result significantly different from the control.

Pancreatic lipase assays were performed to investigate the effects of CSE and GIE on the reduction of fat digestion and absorption. The results indicated that CSE and GIE inhibited lipase activity in a dose-dependent manner (Figure 4). Pancreatic lipase activity was also inhibited by CSE and GIE. The half-maximal inhibitory concentration (IC_50_) values were 2312.44 ± 176.55 *μ*g mL^−1^ and 982.24 ± 44.40 *μ*g mL^−1^, respectively.

### 3.5. FTIR Microspectroscopy

**Figure 5:**
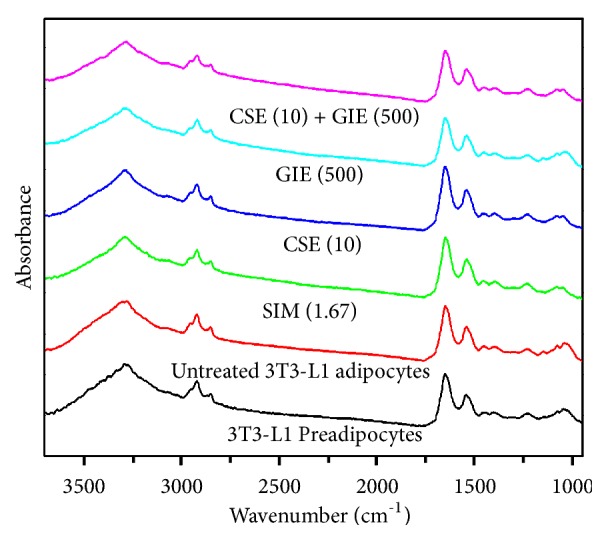
Representative original FTIR spectra (3000–950 cm^−1^) obtained from 3T3-L1 cells at day 10 after differentiation. 3T3-L1 preadipocytes (n = 41); untreated 3T3-L1 adipocytes (n = 39); SIM (1.67) = simvastatin at 1.67 *μ*g mL^−1^ (n = 49); CSE (10) = CSE at 10 *μ*g mL^−1^ (n = 98); GIE (500) = GIE at 500 *μ*g mL^−1^ (n = 76); CSE (10) + GIE (500) = CSE at 10 *μ*g mL^−1^ plus GIE at 500 *μ*g mL^−1^ (n = 97).

**Figure 6:**
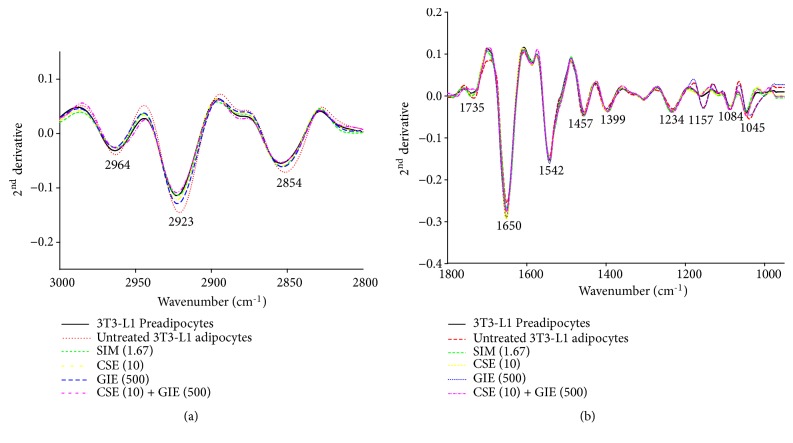
Representative secondary derivative spectra for 3T3-L1 cells. SIM (1.67) = simvastatin at 1.67 *μ*g mL^−1^; CSE (10) = CSE at 10 *μ*g mL^−1^; GIE (500) = GIE at 500 *μ*g mL^−1^; CSE (10) + GIE (500) = CSE at 10 *μ*g mL^−1^ plus GIE at 500 *μ*g mL^−1^. The results represent two regions: (a) lipid (3000–2800 cm^−1^); (b) protein, nucleic acid, glycogen, and other carbohydrates (1800–950 cm^−1^).

The FTIR microspectroscopy technique was used to identify biomolecular changes in groups of preadipocytes that were untreated, exposed to 1.67 *μ*g mL^−1^ SIM, 10 *μ*g mL^−1^ CSE, 500 *μ*g mL^−1^ GIE, or CSE and GIE combined (CSE (10) + GIE (500)) when they were adipocytes on day 10 after differentiation (day 0). Representative FTIR spectra for samples (wavelengths, 3000–950 cm^−1^) are presented in Figure 5. The cells' infrared spectrum profiles for the different treatments were examined for three regions: (1) lipids (3000–2800 cm^−1^), (2) proteins (1700–1500 cm^−1^), and (3) carbohydrates and nucleic acids (1300–950 cm^−1^). Detection of between-cell type differences for the spectra was difficult to achieve using an examination of only the raw spectra. Therefore, they were used for a second derivative analysis (range: 3000–2800 cm^−1^ and 1800–950 cm^−1^) that more clearly and precisely revealed the bands' peak positions (Figures 6(a) and 6(b), resp.). The results for the IR spectra band assignments for the samples are presented in Table 2. They indicated that the spectral differences were mostly in the lipid region (3000–2800 cm^−1^). Strong peaks at 2923 cm^−1^ and 2854 cm^−1^ correspond to CH_2_ asymmetry and symmetric stretching frequency, respectively; both of these were mostly associated with lipids, with some contribution from proteins, carbohydrates, and nucleic acids [30]. The signal intensity increases and increases in the areas of the peaks at 2923 cm^−1^ and 2854 cm^−1^ indicated lipoprotein fractions and the small contributions from carbohydrates and nucleic acids of untreated adipocytes; they were greater than the other peaks (Figure 6(a)) [31–33]. The lipid ester C=O stretching at 1735 cm^−1^ for untreated cells had greater signal intensities and band areas than the other groups (Figure 6(b)). The peak signal intensities and areas for the preadipocytes and the SIM, CSE, and CSE/GIE combination-treated adipocytes at 1157 cm^−1^ indicated absorption peaks for C−O vibrations due to glycogen and other carbohydrates [34]; they were smaller than those for the untreated adipocytes (Figure 6(b)). The signal intensities and areas of the peaks for the preadipocytes and the SIM, CSE, and GIE and CSE/GIE combination-treated groups at 1650 cm^−1^, 1542 cm^−1^, and 1234 cm^−1^ indicated absorption peaks for protein amide I alpha-helix (centered at 1650 cm^−1^), amide II (centered at 1542 cm^−1^), and the functional group of the PO_2_ stretching mode, respectively. The changes of those treated groups were mostly due to changes in nucleic acids, with some contribution from phospholipids (at 1234 cm^−1^) and were greater than the untreated group (Figure 6(b)) [34, 35].

**Figure 7:**
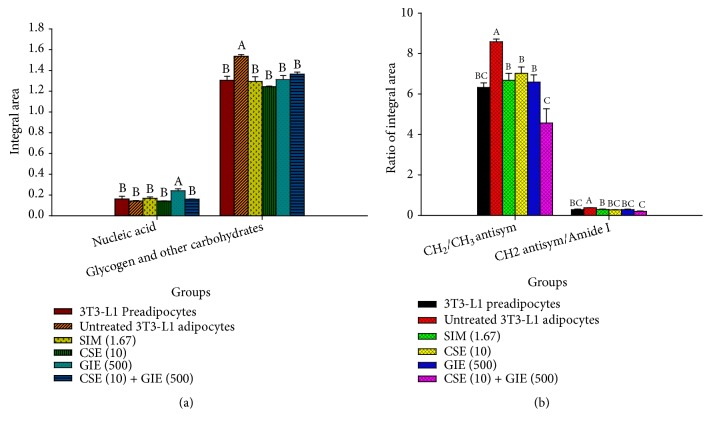
(a) Integrated areas of notable nucleic acids and regions corresponding to glycogen and other carbohydrates and (b) CH_2_/CH_3_ asymmetric stretching and CH_2_ asymmetric stretching/amide I integrated area ratios for 3T3-L1 cells. SIM (1.67) = simvastatin at 1.67 *μ*g mL^−1^; CSE (10) = CSE at 10 *μ*g mL^−1^; GIE (500) = GIE at 500 *μ*g mL^−1^; CSE (10) + GIE (500) = CSE at 10 *μ*g mL^−1^ plus GIE at 500 *μ*g mL^−1^; CH_2_ antisym = CH_2_ asymmetric stretch. The results are presented as mean ± SEM (three replicates). Mean values with the same superscript letter were not significantly different (Tukey's test,* p *< 0.05).

We evaluated changes in macromolecules for preadipocytes and untreated SIM, CSE, GIE, and CSE/GIE combination-treated adipocytes, the integrated areas of the nucleic acid regions (1313–1294 cm^−1^ and 1165–1142 cm^−1^), the regions for glycogen and other carbohydrates (1255–1208 cm^−1^, 1096–1073 cm^−1^, and 1054–1000 cm^−1^), and the ratios for the integrated areas for functional groups associated with lipids and proteins (e.g., CH_2_ (2938–2907 cm^−1^)/CH_3_ (2973–2954 cm^−1^) asymmetric stretching, CH_2_ asymmetric stretching (2938–2907 cm^−1^)/amide I (1673–1624 cm^−1^)) [36]. The results indicated that the integrated areas for the glycogen and other carbohydrate regions in the preadipocyte and SIM, CSE, GIE, and CSE/GIE combination-treated adipocyte groups were significantly smaller than the untreated adipocyte groups. The integrated areas of the GIE-treated adipocyte group nucleic acid regions were significantly greater than the other groups (*p* < 0.05; Figure 7(a)). The values for the ratios of the integrated areas of CH_2_/CH_3_ asymmetric stretching (i.e., associated with the amounts of lipid acyl chain lengths of the lipids) [36] were the greatest for the untreated adipocytes and were significantly greater than the other groups (*p *< 0.05; Figure 7(b)). The statistically significant reduction in the values of the ratios of the integrated areas of CH_2_ asymmetric stretching/amide I in the preadipocytes and the SIM, CSE, GIE, and CSE/GIE combination-treated groups compared with untreated adipocytes (*p* < 0.05; Figure 7(b)) might have been due to alterations in lipid and protein concentrations in each sample. PCA was used for further analysis of the second derivative spectra obtained for the six experimental groups.

Characterization of the spectral properties of biological samples can be achieved using FTIR spectroscopy. The results provide molecular information that varies with macromolecular composition and reflects changes in the absorbance of bands in the FTIR spectrum. IR spectrum molecular fingerprint region absorbance bands in the mid-IR range (4000–400 cm^−1^) are derived from individual structure- and conformation-associated chemical bonds. PCA was used to analyze the characteristics of the biological molecules from the FTIR spectra [29]. This statistical data-reduction method transformed the original values for the variables in the data set into a new set of uncorrected variables (i.e., PCs). We used the PCA to determine the wave numbers present in the complex FTIR spectra that had the statistically significant greatest spectral variation within and between treatment groups. Similar spectra present within the dataset were visualized using scores plots, and variables (the molecular groups within the samples were represented by the spectral bands) were identified using loading plots.

**Figure 8:**
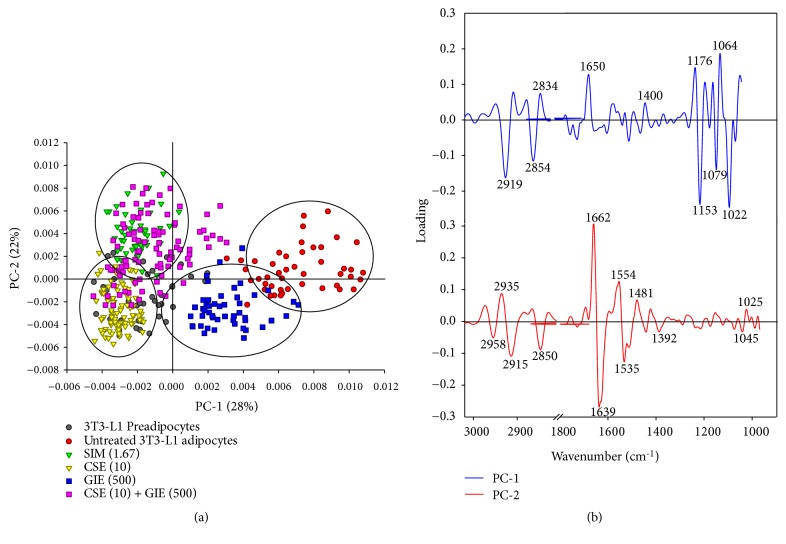
PCA of FTIR spectral ranges 3000–2800 cm^−1^ and 1800–950 cm^−1^. PCA score plot (a) and PCA loading plot (b). Score plots revealed distinct clustering between 3T3-L1 groups. SIM (1.67) = simvastatin at 1.67 *μ*g mL^−1^; CSE (10) = CSE at 10 *μ*g mL^−1^; GIE (500) = GIE at 500 *μ*g mL^−1^; CSE (10) + GIE (500) = CSE at 10 *μ*g mL^−1^ plus GIE at 500 *μ*g mL^−1^. The PC1 and PC2 loading plots identify biomarker differences over a spectral range of samples.

The PCA modeling revealed that distinct clustering of the spectra from the treatment groups was visualized more clearly with the two-dimensional score plot PC1 compared with PC2 (Figure 8(a)). The results from the PCA score plot indicated that the clusters associated with the untreated adipocytes and the GIE-treated group were separated from the clusters associated with the preadipocytes and the SIM- and CSE-treated adipocytes along the PC1 axis (28%). Similarly, the clusters associated with the untreated adipocytes and the SIM-treated group were separated from the GIE- and CSE-treated groups along the PC2 axis (22%). The spectral regions that most contributed to the clustering (Figure 8(a)) were identified using the PCA loading plots (Figure 8(b)). The PC1-associated discrimination was likely associated with the positive loading in the C-H stretch region (centered at 2834 cm^−1^) and the C−O vibrations associated with glycogen and other carbohydrates (centered at 1064 cm^−1^). These characteristics separated the negative score plot of the spectra of the preadipocytes and the SIM, CSE, and CSE/GIE combination-treated adipocytes from the positive score plot of the untreated and GIE-treated adipocytes. Taken together, these results indicated that untreated and GIE-treated adipocytes had greater concentrations of nucleic acids, lipoprotein, lipid acyl chains associated with membrane lipids, and carbohydrates than the preadipocytes and the SIM, CSE, and CSE/GIE combination-treated adipocytes.

The PC2 axis discrimination result between the positive score plot for the spectra associated with untreated adipocytes, SIM, and CSE/GIE combination-treated adipocytes and the negative score plot of the spectra associated with the preadipocytes and CSE and GIE-treated adipocytes can be explained by positive loading of the PC2 axis in the C−H stretch region (centered at 2935 cm^−1^ and at 1662 cm^−1^ (indicating amide I)). There was also negative loading of the PC2 axis in the C−H stretch region (centered at 2915 cm^−1^ and 2850 cm^−1^ and at 1639 cm^−1^ (indicating amide I)).

## 4. Discussion

The doses used in the experiments were standardized based on the results of preliminary screening of concentration intervals. The concentration intervals from the MTT assay that were not toxic and were not significantly different from the control were used in the subsequent experiments. The concentration intervals for the Oil Red O and FTIR assays were obtained from the safety concentration ranges of the MTT assay. The ranges for the lipase inhibition assay were selected from the results of preliminary testing of the tested extracts for the IC_50_ of lipase inhibition activity. The results of this study indicated that during adipocyte differentiation CSE and GIE alone and the CSE/GIE combination induced statistically significant reductions in lipid accumulation. CSE, GIE, and the CSE/GIE combination suppressed lipid accumulation without cytotoxicity. This antiadipogenic effect could have occurred via one or more molecular pathways. Any or all of them might result in lipid accumulation suppression during differentiation.

Treatment of mice with the extract of* C. sinensis *mycelia significantly reduces cholesterol and triglyceride concentrations and the synthesis of very-low-density lipoprotein (low-density lipoprotein precursor) [37, 38]. Therefore, a useful mechanism for obesity prevention might be inhibition of preadipocyte differentiation. Our results revealed that the range in CSE (10–40 *μ*g mL^−1^) that could reduce the lipid accumulation was much lower than the range in GIE (500–2000 *μ*g mL^−1^). The greater effect of CSE on lipid accumulation inhibition may be because CSE has a total flavonoid content and an alkaloid content greater than GIE. CSE also contains cordycepin, which is a main active ingredient that can have strong antiadipogenesis effects. Cordycepin significantly inhibits the biosynthesis of total cholesterol and triglycerides in HepG2 cells [8]; it reduces oleic acid-elicited intracellular lipid accumulation and increases AMPK activity [39].

In patients with diabetes, plasma insulin and glucose level imbalance result in a reduction of triglyceride-derived fatty acid membrane transport; this reduction results in increases in the half-life of triglyceride-rich lipoproteins and remnant particles [40]. There are many suggested mechanisms by which GIE induces its hypoglycemic effect. This mechanism supports the reduction of lipid accumulation [41]. In rats, GIE leaf extracts can inhibit glucose absorption in isolated intestinal tract tissue and suppress the increased blood glucose. The triterpenoid saponin in GIE extracts can suppress the high K^+^-induced contraction of intestinal smooth muscle and affect the Na^+^/K^+^ pump. Pump suppression results in a change in the electrochemical potential of intracellular Na^+^, which affects the Na^+^-dependent cotransport system [7, 42]. Thus, our findings suggested that the mechanism of action of GIE may be inhibition of intestinal tract glucose absorption. The IC_50_ of GIE for the inhibition of pancreatic lipase was 982.24 ± 44.40 *μ*g mL^−1^; previous studies found that orlistat used as a positive control also has this inhibitory effect when used at 68.23 ± 6.67 *μ*g mL^−1^ [21]. Under these conditions, the potential strength of orlistat on lipase activity inhibition is approximately 13.7 times greater than the GIE. Taken together, these results indicate that the inhibitory effects of GIE on pancreatic lipase activity increase in a dose-dependent manner. The enzyme pancreatic lipase has a significant role in fat digestion via hydrolysis of triglycerides to diglycerides and eventually to monoglycerides and free fatty acids [43]. Inhibition of dietary triglyceride absorption via pancreatic lipase inhibition likely represents a new approach for obesity treatment [44]. The lipase inhibition results indicated that the IC_50_ value of GIE was approximately 2.35 times lower than the IC_50_ for CSE. These results are consistent with the results of Hengpratom et al. and Sirichaiwetchakoon et al.;* Oroxylum indicum* extract and* Pluchea indica* tea have lipase inhibition activity, with IC_50_ values of 1062.04 ± 32.21 mg GAE/g and 1708.75 ± 335.85 mg GAE/g of dry weight, respectively [21, 26]. Polyphenolic compounds can inhibit enzyme lipase activity and reduce lipid absorption in the intestine [44, 45]. Hence, the lipase inhibitory effects of both extracts might be dependent on the total phenolic compound concentrations in these extracts; the total phenolic contents in GIE and CSE are 0.81 ± 0.01 mg GAE/g and 0.14 ± 0.01 mg GAE/g of dry weight, respectively.

The antiadipogenic effects of CSE, GIE, and the CSE/GIE combination in 3T3-L1 cells were confirmed by FTIR analysis, which is useful for detection of biomolecular changes in biological samples [29, 46, 47]. We used FTIR analysis to identify spectral profiles of 3T3-L1 preadipocytes and untreated and SIM-, CSE-, GIE-, and CSE/GIE combination-treated 3T3-L1 adipocytes. For the six different conditions, changes in the cells were correlated with changes in the lipid (2964, 2923, and 2854 cm^−1^), nucleic acid (1234 cm^−1^ and 1084 cm^−1^), and glycogen and other carbohydrate regions (1157 cm^−1^ and 1045 cm^−1^) in the second derivative spectra. Compared with the untreated adipocytes, the signal intensities and areas of glycogen and other carbohydrates of the treated adipocytes were significantly smaller after CSE, GIE, or CSE/GIE exposure (*p* < 0.05). Hence, in patients with obesity, decreasing the synthesis of glycogen and other carbohydrates may reduce the rates of carbohydrate and lipid accumulation [48, 49]. The results for the CH_2_/CH_3_ asymmetric stretching ratio indicated that compared with the other groups the untreated adipocytes had significantly greater proportions of longer acyl chains of lipids. The increases in the CH_2_/CH_3_ asymmetric stretching ratio proportions might have been due to lipoprotein fractions involved in lipid storage and accumulation of free cholesterol and cholesterol esters; these fractions can be used as markers of adipogenesis from the preadipocyte to the adipocyte stages [29, 50]. The lipid storage-associated decreases in the CH_2_/CH_3_ asymmetric stretching ratio were also consistent with the Oil Red O staining results. The result for CH_2_ asymmetric stretching/amide I indicated that the untreated adipocyte lipid/protein ratio was significantly greater than the other groups (*p* < 0.05). Taken together, these findings indicated that the lipid/protein results were virtually the same as the lipid storage results.

The classification methods used in this study included PCA, which was used to discriminate the clusters associated with the six FTIR spectra from the different treatments. The discrimination was based on between-group differences in biomolecular composition (nucleic acid, amide I protein, lipids, and glycogen and other carbohydrates). Significant differences between the spectra from the six sample groups were examined using a two-dimensional score plot obtained from the PCA (Figure 8(a)). The loading plots revealed relationships among the six sample groups for variables that contributed to clustering (Figure 8(b)). The score plot revealed that the untreated adipocyte and GIE-treated adipocyte clusters were separate from the clusters associated with the other four groups along the PC1 axis and accounted for 28% of the total variance. Twenty-two percent of the total variance was described with the PC2 score plot; the clusters associated with the untreated adipocytes were clearly separated from the clusters associated with the GIE-treated adipocytes. The discrimination along PC1 between the negative score plot for the preadipocytes, SIM, CSE, and CSE/GIE combination-treated adipocyte spectra and the positive score plot for the untreated and GIE-treated adipocyte spectra can be explained by the negative PC1 loadings for the preadipocytes and the SIM, CSE, and CSE/GIE combination centered at 2950 cm^−1^, 2854 cm^−1^, 1153 cm^−1^, and 1022 cm^−1^ and the positive loading of the other two groups centered at 2834 cm^−1^, 1650 cm^−1^, and 1064 cm^−1^ (Figure 8(b)). Similarly, the PC2 loading between the positive score plot centered at 2935 cm^−1^ and 1662 cm^−1^ for the untreated adipocytes and the negative score plot centered at 2915 cm^−1^, 2850 cm^−1^, and 1639 cm^−1^ for the GIE-treated adipocytes was apparent. Taken together, these results indicated that this approach could be adapted for the obesity biomarker development and evaluation of the efficacy of obesity drugs in patients with obesity.

## 5. Conclusions

In summary, the flavonoids were the important group of phytochemical compounds found in both CSE and GIE; alkaloids were found only in CSE. The viability of cells treated with CSE (40) or GIE (2000) alone or combined (CSE (10) + GIE (500)) was not significantly reduced compared with the preadipocytes (*p* > 0.05). The Oil Red O and hematoxylin staining results indicated that compared with no treatment the combination of CSE (10) and GIE (500) resulted in synergistic effects on the reduction in adipocyte lipid accumulation and significantly decreased lipid accumulation (*p *< 0.05). The FTIR assay revealed that adipocytes treated with CSE (10) plus GIE (500) had significantly lower lipid levels than the adipocytes treated with CSE (10) or GIE (500) alone and the adipocytes that were untreated (*p *< 0.05). The ratios of the integrated areas of glycogen and other carbohydrates and lipid/protein ratios in the adipocytes treated with CSE (10) or GIE (500) alone or combined were significantly lower than those for the untreated adipocytes (*p* < 0.05). These findings indicated that the use of FTIR microspectroscopy combined with the multivariate statistical PCA could be an effective approach for classification of differentiated adipocytes.

## Figures and Tables

**Table 1:** Preliminary qualitative phytochemical screening of *C. sinensis* and *G. inodorum* extracts.

Phytochemical compounds	*Cordyceps sinensis*	*Gymnema inodorum*
Alkaloids	*+*	*-*
Flavonoids	*+*	*+*
Tannins	*+*	*-*
Terpenoids	*-*	*+*
Saponin	*+*	*-*
Glycoside	*-*	*+*
Steroids	*-*	*-*

(+) = presence; (-) = absence

**Table 2:** Fourier transform-infrared band assignments.

Second derivative spectra band position (cm^−1^)	Band assignments
2964	CH_3_ asymmetric stretch associated with membrane phospholipid methyl terminals: mainly lipid
2923	CH_2_ asymmetric stretch associated with membrane phospholipid methylene groups: mainly lipids, some contribution from proteins, carbohydrates, and nucleic acids
2854	CH_2_ symmetric stretch: mainly lipids, some contribution from proteins, carbohydrates, and nucleic acids
1735	C=O stretching vibration: lipids (triglycerides and cholesterol esters)
1650	Amide I: C=O (80%) and C−N (10%) stretching, N−H (10%) bending vibration: *α*-helix proteins
1542	Amide II: N−H (60%) bending and C−N (40%) stretching vibration: *α*-helix proteins
1457	CH_2_ bending vibration: lipids and proteins
1399	COO-symmetric stretching and CH_3_ bending vibration: lipids, proteins
1234	PO_2_-asymmetric stretching vibration: RNA, DNA, and phospholipids
1084	PO_2_-symmetric stretching vibration: RNA, DNA
1157	C−O vibration: glycogen and other carbohydrates
1045	C−O vibration: glycogen and other carbohydrates

## Data Availability

The datasets used and analyzed during this study are available from the corresponding author on reasonable request.
